# Eyes, Menstruation and Endometriosis

**DOI:** 10.52054/FVVO.15.2.074

**Published:** 2023-06-30

**Authors:** S Rahman, Y Youssef, G Maroun, D Inaty, M.H. Kheil, G Moawad

**Affiliations:** Department of Obstetrics and Gynecology, George Washington University, Washington, DC, USA; Department of Obstetrics and Gynecology, Hurley Medical Center/Michigan State University College of Human Medicine, Flint, MI; Faculty of Medicine, University of Balamand, El Koura, Lebanon; Department of Pathology, Wayne State University, Detroit, MI, USA; The Center for Endometriosis and Advanced Pelvic Surgery, Washington, DC, USA

**Keywords:** Haemolacria, vicarious menstruation, ocular endometriosis, bleeding, tears, oestrogen

## Abstract

Vicarious menstruation refers to cyclical bleeding outside the uterine cavity during the menstrual cycle. Haemolacria, or blood in tears, is a rare medical phenomenon that can occur with menstruation or in association with endometriosis. Endometriosis, defined by the presence of endometrial-like tissue in extra-uterine sites, affects around 10% of fertile women; the ocular system is one of the rarest sites it can be present in. Endometriosis usually requires a biopsy for diagnosis, and the anatomic difficulty of obtaining a biopsy of the ocular system makes ocular endometriosis diagnosis more obscure. However, few cases have been described in the literature and due to the psychological, physical, and social implications of haemolacria on the patient, treatment is of utmost importance. We reviewed the literature on ocular endometriosis and ocular vicarious menstruation with the aim to discuss the clinical presentation, necessary workup and various treatment modalities, while also shedding light on the connection between the eyes and endometriosis in general. It is hypothesised that uterine endometrial cells can travel lymphatically or haematogenously and deposit extra-uterine endometriotic lesions that bleed in response to hormonal changes in the menstrual cycle. Additionally, the conjunctival vasculature has been found to be responsive to hormonal changes due to the presence of oestrogen and progesterone receptors, causing bleeding at the corresponding sites, even without endometriotic lesions. Clinical correlation of the haemolacria with the menstrual cycle can suffice for a diagnosis of vicarious menstruation, and thus opens the possibility of treatment to provide symptomatic treatment for the patient.

## Introduction

Almost all reproductive-aged females worldwide menstruate monthly after menarche, and normally this is limited to uterine bleeding. Vicarious menstruation represents a rare condition that affects post-menarche females where menstrual shedding is not only occurring in the uterus; it is bleeding that occurs in concordance with the menstrual cycle in extragenital organs and is suppressed with ovulation suppression drugs or physiological states like pregnancy. Vicarious menstruation is most often diagnosed in the third decade of life, often concomitantly with pelvic endometriosis (Barat and Kwedar, 1988). It usually manifests itself in younger women that have not yet reached the menopausal or perimenopausal phase (Murube, 2011). Substitutive vicarious menstruation refers to extragenital bleeding occurring in the absence of genital bleeding. Supplementary vicarious menstruation occurs in most cases and refers to concomitant extrauterine and normal menstrual bleeding (Abboud and Hanna, 1971). Vicarious menstruation has been most reported in the nasal mucosa, but additionally in the lungs, stomach, intestine, kidney, lips, skull, skin, and eyes (Barat and Kwedar, 1988; Israel, 1941). Extragenital bleeding that occurs cyclically in an amenorrhoeic woman or that precedes normal menstruation does not exclude vicarious menstruation (Barat and Kwedar, 1988).

Endometriosis is defined by the presence of endometrial-like tissue outside the uterine cavity. Extra-pelvic endometriosis that leads to extra-genital bleeding can often be a cause of vicarious menstruation (Türkçüoğlu et al., 2008). Endometriosis affects around 10% of women, and it is unfortunately often underdiagnosed. This condition has significant implications on patients’ quality of life and can range from severe cyclic pelvic pain to infertility. Recognising clinical symptoms of endometriosis can lead to diagnosis and management optimisation, all of which are crucial to the patient’s wellbeing (Türkçüoğlu et al., 2008).

It is presumed that in 5% of endometriosis cases, endometrial tissue can reach distant organs lymphatically and haematogenously (Oner et al., 2006). Rarely, extra-pelvic endometriosis can occur in the eyes and cause ocular bleeding. A case series of vicarious menstruation by Roth in 1920 reported ocular symptoms in only 1% of patients (Barat and Kwedar, 1988). Because of its challenging presentation and psychosocial effects on the patient, research into ameliorating treatment modalities and prophylactic prevention is critical. This narrative review aims to present all ocular endometriosis and vicarious menstruation cases reported in the literature, describe different presentations, and discuss various treatment modalities.

## Materials and Methods

A comprehensive review of the literature was carried out for English publications in PubMed, Medline, and Google Scholar, including all relevant studies found using the following Medical Subject Headings (MeSH) terms and keywords: ocular endometriosis; ophthalmic endometriosis; nasolacrimal endometriosis; ocular vicarious menstruation; conjunctival haemorrhage; haemolacria; or eyes and endometriosis.

The search resulted in a total of 7,772 articles across different databases. Articles in languages other than English were excluded, and the article type was limited to case reports and case series. Abstracts were skimmed and duplicates were removed. A more detailed inspection of the papers allowed exclusion of articles in which haemolacria was not correlated to the menstrual cycle.

**Figure 1:**
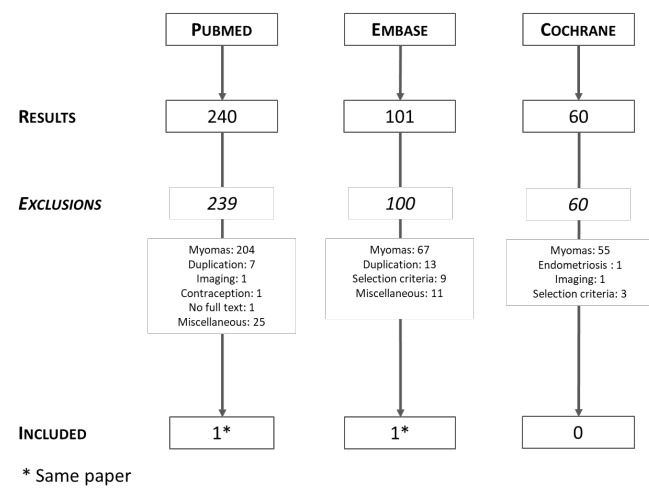
Flow diagram of the literature search. * Estadella et al., 2018.

## Reported cases

### i) Ocular Vicarious Menstruation

In 1581, Dodanaeus described the first case of ocular vicarious menstruation (Barat and Kwedar, 1988). To date, the pathophysiology of this condition remains unclear; however, it has been hypothesized that hormonal changes in oestrogen and progesterone levels during the menstrual period increase vascular permeability. This leads to extravasation and bleeding at the corresponding extra-uterine sites (Dunn, 1972; Javert, 1951).

Abboud and Hanna 1971 described a case of a 17-year-old female who reported bleeding from her eyes and occasionally from her nose that had started 8 months prior (Abboud and Hanna, 1971). The bleeding episodes coincided with her menstrual cycle, except for one incident that occurred independently. The patient’s history was also notable for jaundice and epilepsy. On presentation, the patient was actively experiencing a bleeding episode. Ophthalmologic examination revealed no conjunctival vessel congestion, no disturbances in visual acuity or abnormal lesions. Radiologic imaging was normal, and blood work revealed anaemia. No direct indicative cause could justify the bleeding that was seeping from the conjunctival sac, but its cyclic pattern was enough to correlate it to vicarious menstruation (Abboud and Hanna, 1971).

Another case report by Barat and Kwedar (1988) described a 17-year-old girl who experienced intermittent bleeding from her right eye during her menstrual cycle. These bleeding episodes occurred about 10 times per day, each lasting two to three minutes. They began with retro- orbital pain and pressure that ceased once her eye started bleeding. She also experienced right-sided headaches that were relieved by acetaminophen and codeine. She denied any visual disturbances, such as diplopia or photophobia, denied any olfactory disturbances, cheek numbness, sinus pain, temporomandibular joint pain or epistaxis. On examination, her vision was normal in the left eye, and 20/100 in the right eye (must be as close as 20 feet to see what a person with normal vision can see at 100 feet). Full ophthalmological examination revealed no abnormalities in the pupils, intraocular pressure, discs and maculae. Blood was seen flowing from the fornix, medial canthal region and lacrimal gland area. No tumours were seen in the conjunctiva or lacrimal glands, and the slit lamp exam was normal. Dacryocystogram and IV fluorescein injection and examination under cobalt blue light eliminated any capillary abnormality with no masses seen. Review of her gynaecologic, neurologic, otolaryngologic and psychiatric systems was normal. Laboratory tests, skull and face X-rays, head and neck computerised tomography (CT) scan with contrast, bilateral carotid angioscopy, electroencephalography (EEG) and spinal tap did not reveal any abnormal findings. Bleeding disorders were ruled out. Endometrial cells were not seen on cytological examination of the bleeding. The diagnosis of ocular vicarious menstruation was made based on the temporal timing and the catamenial occurrence (Barat and Kwedar, 1988).

Dua et al. (2014) presented the case of a 31-year- old African American woman with past medical history of hypertension, who experienced right- sided orbital and subconjunctival haemorrhagic episodes of 7 days duration, coinciding with menstruation for the past year. Like the other clinical cases, laboratory tests, ophthalmic and radiologic workups eliminated other aetiologies. While the patient had elevated antinuclear antibodies, anti-SSA antibodies and anti-ds DNA antibodies, there was not sufficient evidence to diagnose her with systemic lupus erythematosus. She did not have pertinent past family history or personal precipitating factors. The patient was clinically diagnosed with orbital vicarious menstruation based on her cyclical symptoms. She reported improvement after treatment with oral contraceptive pills containing levonorgestrel/ ethinyl oestradiol at a 4-months follow-up visit (Dua et al., 2014).

The most recent reported case of ocular vicarious menstruation is of a 25-year-old woman who presented for bilateral haemolacria (Ghosh et al., 2021). This was her second episode of such symptoms; the first being a month prior to presentation with epistaxis. Neither episode was associated with pain or headache. All diagnostic workup was normal, eye injuries were eliminated, and no family history of haemolacria was reported. Her episodes occurred concomitantly with her menstrual cycles. The authors reasoned that the cyclical hormonal levels of oestrogen and progesterone that occur, caused increased ocular vascular permeability leading to bleeding from these capillaries. Moreover, it is worthy of note that the cornea is hormone-responsive, and its thickness and curvature vary in different physiological cases like menstruation, pregnancy and lactation, possibly providing an outlet for blood (Ghosh et al., 2021).

**Table I:** Reports of Ocular Vicarious Menstruation.

Authors	Year	Reported Case
Barat and Kwedar	1988	13-year-old girl with recurrent ocular bleeding during menstruation. Bleeding was localized to the conjunctiva and subsided with hormonal therapy.
Abboud & Hanna	1971	5 cases of spontaneous bleeding from the bulbar conjunctiva in adolescent girls, all occurring during menstruation. The bleeding stopped spontaneously within 1-2 days.
Dua et al.	2014	22-year-old woman presenting with unilateral ocular bleeding during menstruation. Imaging revealed an orbital varix, which was treated with embolization.
Ghosh et al.	2021	25-year-old woman presenting with bilateral ocular bleeding during menstruation. No underlying pathology was identified, and the bleeding resolved spontaneously within 2 days.

**Table II:** Reports of Ocular Endometriosis.

Authors	Year	Reported Case
Sharghi et al.	2019	A case of endometriosis of the eyelid in a 32-year-old woman presenting as a cutaneous nodule. Histopathological examination revealed endometrial tissue.
Oner et al.	2006	A case of nasolacrimal endometriosis in a 26-year-old woman presenting with recurrent bloody tears during menstruation. Imaging revealed a soft tissue mass in the left nasolacrimal duct, which was excised. Histopathological examination confirmed the diagnosis of endometriosis.
Türkçüoğlu et al.	2008	A case of presumed nasolacrimal endometriosis in a 27-year-old woman who presented with bloody tearing during menstruation. Imaging revealed a soft tissue mass in the left nasolacrimal duct, which was excised. Histopathological examination revealed a fibrous tissue with hemosiderin deposits but no endometrial tissue.
DeRuyter et al.	2022	A case of ectopic endometriosis of the eyelid in a 26-year-old woman. Histopathological examination revealed endometrial tissue.

### ii) Ocular Vicarious Menstruation associated with Endometriosis

Sharghi et al. 2019 reported the first known case of cutaneous endometriosis on the eyelid. A 41-year- old woman presented with an asymptomatic non- blanching telangiectatic macule of six months duration on her right upper eyelid. The patient did not report any bleeding, increase in pain or macule size in concordance with her menstrual cycle. Histological analysis of the lesion biopsy showed endometrial glands and stroma. Immunohistochemistry showed a strong expression of CD10 as well as oestrogen and progesterone receptors, similar to immunohistochemistry of a uterine endometrial biopsy (Sharghi et al., 2019). Like vicarious menstruation, the pathophysiology of cutaneous endometriosis remains unclear; the most likely hypothesis currently is metastatic endometrial cells (Sharghi et al., 2019; Alnafisah et al., 2018). Important to note, this patient also reported irregular and painful menstrual cycles, suggestive of pelvic endometriosis (Sharghi et al., 2019). Cutaneous endometriotic nodules are usually excised irrespective of whether hormonal therapy like danazol and leuprolide was used pre- operatively to decrease their size (Sharghi et al., 2019; Puravis and Tyring, 1994).

Oner et al. 2006 reported a case of a 28-year- old woman with cyclical haemolacria of 14 years duration from the puncta of her eyes. Ophthalmological examination, cytological evaluation of nasolacrimal lavageand haematological workup showed no abnormalities. Due to anatomic difficulty, a biopsy was not performed. Again, the leading diagnosis was nasolacrimal endometriosis based on clinical presentation. Interestingly, the patient reported that when she became pregnant, her bleeding episodes ceased (Oner et al., 2006).

In a case report, Türkçüoğlu et al. 2008 described the case of a 13-year-old female who presented withcyclic bleeding from the left inferior punctum that coincided with her menstrual cycle. Her episodes began with the onset of menarche one year prior. Clinically, crusted haemorrhage was seen in the left tear meniscus. No endometrial cells were found on nasolacrimal lavage. Gradient echo magnetic resonance imaging (MRI) performed during menstruation showed a hypodense haemorrhagic tissue in the nasolacrimal canal. This led to the diagnosis of nasolacrimal endometriosis, after the elimination of dacryocystitis and solid lacrimal sac tumours, which would usually appear as hyperintense lesions (Türkçüoğlu et al., 2008). A rebuttal to this diagnosis and the clinical case suggested that the presumed nasolacrimal endometriosis could have been a hobnail haemangioma (Brown et al., 2009). However, the original authors refuted this claim by comparing the case to a previously diagnosed haemangioma that presented roughly the same way minus the bleeding, which was usually a marker of endometriosis when triggered by hormonal changes(Brown et al., 2009).

While ocular vicarious menstruation mostly occurs pre-menopause, DeRuyter et al. (2022) reported a case of a 57-year-old postmenopausal woman with a history of endometriosis on hormonal replacement therapy, presenting with a papillomatous lesion on her left lower eyelid margin. Histological study post-excisional biopsy showed poorly preserved glandular epithelium with atypical large nuclei, hyperchromatic and mitotic figures staining positive for pan-cytokeratin, PAX8 and oestrogen receptor. Stromal cells showed positive CD10 staining. Her pelvic MRI was normal. Her past surgical history was significant for endometrial ablation, subsequent laparoscopic supracervical hysterectomy and left salpingo- oophorectomy. After ruling out the possibility of an endometrial malignancy, the favoured diagnosis was extra-pelvic endometriosis (DeRuyter et al., 2022).

## Endometriosis and the eye

The relationship between the ocular system and endometriosis is not limited to a few reports of ocular endometriosis; there have been studies showing unconventional associations between endometriosis and the eyes. A study conducted by Vercellini et al. (2014) aimed to find a correlation between eye colour and endometriosis. They reviewed the many previous studies conducted that showed that women with deep infiltrating endometriosis share common phenotypic characteristics (Viganò et al., 2012; Lafay Pillet et al., 2012). Vercellini et al. (2014) found a clear correlation between having blue- coloured eyes and deep infiltrating endometriosis.

Previous understanding of the condition was that the growth of vicarious endometriosis was fuelled by hormones or factors released by the endometrial tissue itself or from environmental factors in the peritoneum (Turan et al., 2020). In 2016, Silveira and colleagues described implanted rat endometrial tissue in a non-peritoneal environment to test that assumption (Turan et al., 2020). They transplanted the endometrial tissue into the anterior eye chamber, following the works of those before them. Surely enough at 3 and 6 weeks post-transplant, the endometrial allografts showed sympathetic and sensory innervation whilst retaining the histochemical characteristics of the original tissue. This not only proved the possibility of endometrial tissue having its own neurogenic capabilities but also showed that there could be environmental factors within the eye chamber that can maintain endometrial growth (Turan et al., 2020).

In an animal model, other researchers used the immunologically privileged anterior chamber of ovine eyes in 12 sheep to implant human endometrial curetting (Garry, 2010). They observed that almost a week after the transplant, the implants developed novel intrinsic and extrinsic blood supplies and new surface epithelium. Some of the implants exhibited new gland growth and were able to invade the iris and cornea. This model of endometrial growth can provide the means for researchers to study seeding and growth of endometrial cells in extra-uterine locations (Garry, 2010).

Beyond the spectrum of animal models, a further connection was established between endometriosis and the eye. Previous studies had suggested that the oestrogen released by the endometrial cells could inhibit the function of the meibomian gland leading to inflammation (Mantelli et al., 2007). Turan et al. (2019) aimed to study the effect of endometriosis on the ocular surface. They utilised non-invasive conjunctival impression cytology (CIC) to study the changes of endometriosis at the cellular level (Gulay et al., 2019). They also used Schirmer 1 and tear breakup time tests to further evaluate tear film quality. The results of the study showed a significant correlation between the presence of endometriosis and a decrease in tear production and quality. In addition to that, the CIC scores showed a significant drop in goblet cell counts as well as a rise in squamous metaplasia in the conjunctival epithelium (Turan et al., 2020).

Matalliotakis et al. (2017) reviewed visual symptoms of 900 women with endometriosis, 55 of whom were adolescents. Ten patients were found to have varying vision and a sensation of a foreign body in the eye. Based on the minimal evidence of nasolacrimal endometriosis in the literature, they opted to perform eye examinations and diagnosed these patients with dry eye syndrome (Oner et al., 2006; Türkçüoğlu et al., 2008). This supports the hypothesis that sex hormones play a role in the aetiology of dry eye syndrome, thus highlighting the logical path in which endometriosis is linked to or is itself involved in ocular pathology (Sullivan, 2004).

A study conducted by Ottovay and Norn (1991) was designed to detect a correlation between haemolacria and menstruation without gross bloody tearing. This study was partly based on the early descriptions of a correlation between haemolacria and endometriosis in older studies. They gathered 125 healthy participants and used the Stix method to determine whether their tears contained traces of blood. Blood was noted in the tears of 18% of fertile women in their menstrual phase, 7% of pregnant women, and in none of the menopausal women. While suggesting that occult haemolacria may normally occur in a percentage of menstruating women, they mentioned that it most frequently occurs during phase III of menstruation or the ‘spotting’ phase. This may suggest that a menstruation-like occurrence could exist in the conjunctiva (Ottovay and Norn, 1991).

## Diagnosis

Haemolacria occurs mostly in females, mainly unilaterally, and is treated expectantly if all other aetiologies are eliminated (Ambasta et al., 2021). Before the diagnosis of ocular endometriosis or ocular/orbital vicarious menstruation can be made, imaging studies need to rule out other lesions or masses, both in the lacrimal and vascular systems (Barat and Kwedar, 1988). Patients need to be asked about the history of any trauma, hypertension, use of any anticoagulants, and a concomitant presentation of epistaxis and its temporal relation to haemolacria (Barat and Kwedar, 1988; Dua et al., 2014). Complete blood count needs to be done to rule out anaemia, as it is a possible cause of haemolacria (Abboud and Hanna, 1971). The possible presence of collagen, vascular or blood disorders also needs to be eliminated (Barat and Kwedar, 1988). Any source of ocular, orbital, cutaneous, or nasolacrimal inflammation or infection may cause haemolacria and needs to be ruled out (Ambasta et al., 2021). A comprehensive differential for a potential eyelid mass or haemolacria can include chalazia (styes), milia, xanthelasma and even basal cell carcinoma. Bleeding disorders, such as haemophilia, melanoma, and even pyogenic granulomas can cause haemolacria (Pe’er, 2016; Stokkermans and Prendes, 2022).

According to Türkçüoğlu et al. 2008, the gold standard to diagnose endometriosis in extra-genital organs is through a biopsy and histological study. However, some sites, such as the nasolacrimal canal, make obtaining a biopsy anatomically difficult. Moreover, in some cases, a cytological examination from lavage did not provide any findings related to endometriosis (Türkçüoğlu et al., 2008). Therefore, for diagnosis, physicians must resort to the clinical presentation of catamenial cyclical extra- genital bleeding coinciding with menstruation. As mentioned previously, the presence of confirmed endometriosis, especially with extra pelvic disease (diaphragmatic lesions, pulmonary, CNS, etc) is an important clinical clue to the possibility of ocular endometriosis (Stokkermans and Prendes, 2022; Tripathy and Salini, 2022).

## Treatment

Since ocular endometriosis and vicarious menstruation are rare phenomena, there is no definitive established treatment. Additionally, treatment is tailored based on the patient’s preferences. Treatment options for vicarious menstruation are similar to medical treatments for pelvic endometriosis; they include ovulatory suppression with hormonal birth control pills or endometrial tissue suppression with Danazol which blocks the release of gonadotropins. Surgery is an option if the endometrial tissue is in the anatomically accessible area (Barat and Kwedar, 1988). Additionally, since it is believed that increased capillary permeability that occurs during the menstrual cycle leads to bleeding, strengthening the capillary walls with vitamins K, C or P could render beneficial results (Abboud and Hanna, 1971).

Enovid, a hormonal birth control pill that is now discontinued, subsided the bleeding when administered to the patient in Barat and Kwedar’s study. The oestrogen therapy proved beneficial in her treatment (Barat and Kwedar, 1988). Even though ocular endometriosis was not stated as a definitive diagnosis in this case, all other differentials were ruled out, and so the bleeding was established as vicarious menstruation. It was hypothesised that the bleeding was due to the ability of the cyclical levels of oestrogen and progesterone to increase vascular permeability in extra-genital tissues (Dunn, 1972).

Since the bleeding was not reported to be bothersome to the 13-year-old patient in Kurt et al.’s study, the authors resorted to expectant management in hopes that with time, her cycles would become less anovulatory, and therefore could reverse her vicarious menstruation. If immediate management was required, hormonal therapy would have been the treatment of choice (Türkçüoğlu et al., 2008). In the most recent case, a patient who was started on a combination of oral contraceptive pills achieved cessation of bleeding episodes at a three-month follow up. (Ghosh et al., 2021).

Fowler et al. (2015) decided to experiment with a new technique where they used punctal plugs to treat their patients. Both patients reported a relief from bloody tearing with a rapid decrease in frequency and an eventual cessation along with a huge psychological reprieve. Given that ocular endometriosis was never ruled out in those patients, and that punctual plugs are not a disease specific treatment, there could be a chance for them to be considered as a treatment option in future cases.

## Conclusion

Women can experience countless gynaecologic diseases in their pre-menopausal years, and endometriosis can affect up to 15% of these women. Ocular endometriosis is a rare, underdiagnosed subtype, with no established pathogenesis, diagnostic criteria or means of treatment. A thorough review of the available literature has shown that the anatomic location of ocular endometriosis renders it even harder to diagnose, preventing physicians from obtaining a biopsy for a definitive diagnosis or excising it to provide treatment. Moreover, the eye was shown to be responsive to hormonal changes occurring in the menstrual cycle, which cease to occur in physiologic states like pregnancy or exogenous ovarian suppression regimens. Endometrial tissue is also hypothesised to travel lymphatically or hematogeneously to reach extra-pelvic organs like the eye. After eliminating all other possible aetiologies of haemolacria with full radiologic and laboratory workup, vicarious menstruation in the presence or absence of ocular endometriosis is a clinical diagnosis based on the correlation with the timing of menstruation. The patient could be managed expectantly or with hormonal treatments based on the patient’s preference.
